# High-Risk Intracranial Atherosclerotic Stenosis Despite Aggressive Medical Treatment: Protocol for a Prospective Nested Case-Control Study

**DOI:** 10.3389/fneur.2022.803224

**Published:** 2022-04-13

**Authors:** Tao Wang, Jichang Luo, Changyi Liu, Bin Yang, Ran Xu, Long Li, Kun Yang, Chao Zhang, Yabing Wang, Yanfei Chen, Peng Gao, Jian Chen, Liqun Jiao, Yan Ma

**Affiliations:** ^1^China International Neuroscience Institute (China-INI), Beijing, China; ^2^Department of Neurosurgery, Xuanwu Hospital, Capital Medical University, Beijing, China; ^3^Chinese Academy of Medical Sciences, Peking Union Medical College, Beijing, China; ^4^Department of Evidence-Based Medicine, Xuanwu Hospital, Capital Medical University, Beijing, China; ^5^Beijing Escope Technology Inc, Beijing, China; ^6^Department of Interventional Radiology, Xuanwu Hospital, Capital Medical University, Beijing, China

**Keywords:** intracranial atherosclerosis, medical treatment, vessel wall, hemodynamics, protocol

## Abstract

**Introduction:**

Intracranial atherosclerotic disease (ICAD) is one of the most important etiologies of ischemic stroke, especially in Asia. Although medical treatment was recommended as the first-line therapy for ICAD, the recurrent stroke rate was still high in severe stenosis of ICAD despite aggressive medical treatment. Traditionally, the degree of luminal stenosis is used as the principal index for stroke risk stratification in patients with ICAD, while recent evidence suggested that symptomatic atherosclerotic plaques were characterized by plaque features and hemodynamics. This prospective, longitudinal, and nested case-control study aims to identify multimodal imaging predictors of high-risk patients with ICAD refractory to medical treatment and explore a refined risk stratification model based on the above multimodal imaging predictors.

**Methods:**

This prospective, longitudinal, and nested case-control study includes 400 symptomatic patients with ICAD with 50–99% of stenosis treated with aggressive medical therapy. All patients who meet the eligibility criteria are assessed by multimodal imaging examination from three aspects, including lumen stenosis, plaque characteristics, and hemodynamic features. The enrolled patients receive aggressive medical management, including antiplatelet therapy and cardiovascular risk control. The primary outcome is ischemic stroke or death attributable to the lesion of the target vessel within 1 year. The secondary endpoints are (1) any stroke or death; (2) all-cause mortality; (3) any stroke out of the territory of the responsible lesion; (4) functional outcome with the modified Rankin Scale (mRS).

**Ethics and Dissemination:**

This study has been approved by the ethics committee of our center ([2021]083) and has been prospectively registered (Registration No: ChiCTR2100048832). Study findings will be disseminated through peer-reviewed publications and presentations at scientific meetings.

## Introduction

Intracranial atherosclerotic disease (ICAD) is one of the most important etiologies of ischemic stroke, especially in Asia. Approximately 33–50% of cases of stroke and more than 50% of transient ischemic attacks (TIA) are attributed to ICAD, which is the most common cause of stroke burden in China (1–4). Contrary to Caucasian patients, patients in Asia with ICAD have a high occurrence rate of severe stenosis and multiple risk factors, such as hyperlipidemia, diabetes mellitus, a family history of stroke, smoking, heavy drinking, and overweight (5–7).

The primary treatments for ICAD are medical treatments and endovascular treatments. Endovascular treatments, such as primary angioplasty, balloon-mounted stent, and self-expansion stent, were regarded as a substitution for patients refractory to medical treatment (8). According to previous randomized control trials studies, endovascular treatment for symptomatic ICAD was related to a higher recurrent risk of stroke (19.7–36.2%) than medical treatment (9, 10), while it may provide benefits over medical treatment with tailored patient selection and operator credentialing (11, 12). Medical treatment, comprising antithrombotic therapy, lipid-lowering treatment, and risk factor modification, was recommended as the first-line therapy for ICAD since the Stenting vs. Aggressive Medical Therapy for Intracranial Arterial Stenosis (SAMMPRIS) trial suggested that medical treatment was superior to the endovascular treatment. However, the recurrent stroke rate ranged from 12.2 to 23% per year in patients with severe stenosis of ICAD even under aggressive medical treatment (8–10, 13, 14).

A great deal of effort has been invested in early identification of high-risk patients with ICAD despite aggressive medical treatment for alternative therapy, such as endovascular therapy, but with limited effect. Traditionally, the degree of luminal stenosis referred to the Warfarin-Aspirin Symptomatic Intracranial Disease (WASID) criteria is used as the principal and is somehow the only index for stroke risk stratification in patients with ICAD, which is simple but may not be sufficiently distinguishable (15). Recent evidence suggested that symptomatic atherosclerotic plaques were characterized not only by a severe degree of luminal stenosis but also by the features such as plaque instability and hemodynamic compromise (16).

Plaque features including specific plaque components, such as large lipid core, thin fiber cap, incomplete fiber cap, intra-plaque hemorrhage, and inflammatory cell infiltration around the lesions, have been collectively characterized as “plaque instability,” which has been demonstrated to be associated with risk of distal embolization or cerebral blood flow deficits (17–19). High-resolution MRI (HR-MRI), which is an emerging effective non-invasive technique, has been used to assess the above features in practice. For instance, intra-plaque hemorrhage and plaque enhancement were detected on HR-MRI in more than 1/2 and 2/3 of patients with ischemic stroke caused by ICAD, respectively (20–22). Hemodynamic compromise, namely hypoperfusion, is another important dimension of ICAD assessment and would impair the washout of debris during distal intracranial circulation, leading to artery-to-artery embolism infarction and increasing the risk of ischemic events (23). Studies have reported that the hazard ratio of ischemic stroke in ICAD patients with hemodynamic compromise was higher with a range of 2–3.5 compared with ICAD patients without hemodynamic compromise after adjusting age and other cardiovascular risk factors (24–27). Cerebral hypoperfusion could be evaluated by several imaging techniques, including CT perfusion, perfusion-weighted imaging, and arterial spin labeling (ASL). In contrast to CT perfusion and perfusion-weighted imaging, ASL is a non-invasive magnetic resonance perfusion imaging method used to visualize and quantify cerebral bleed flow without exogenous contrast agents. Computational fluid dynamics (CFD) simulation is an effective supplement to ASL for hemodynamic evaluation, which could be applied for the interpretation of local flow properties such as translesional pressure drop, wall shear stress, and filtration rate of low-density lipoprotein (28–30). In this study, we have described the methods and procedures used in this prospective, longitudinal, and nested case-control study.

## Methods and Analysis

### Study Design

**Figure 1:**
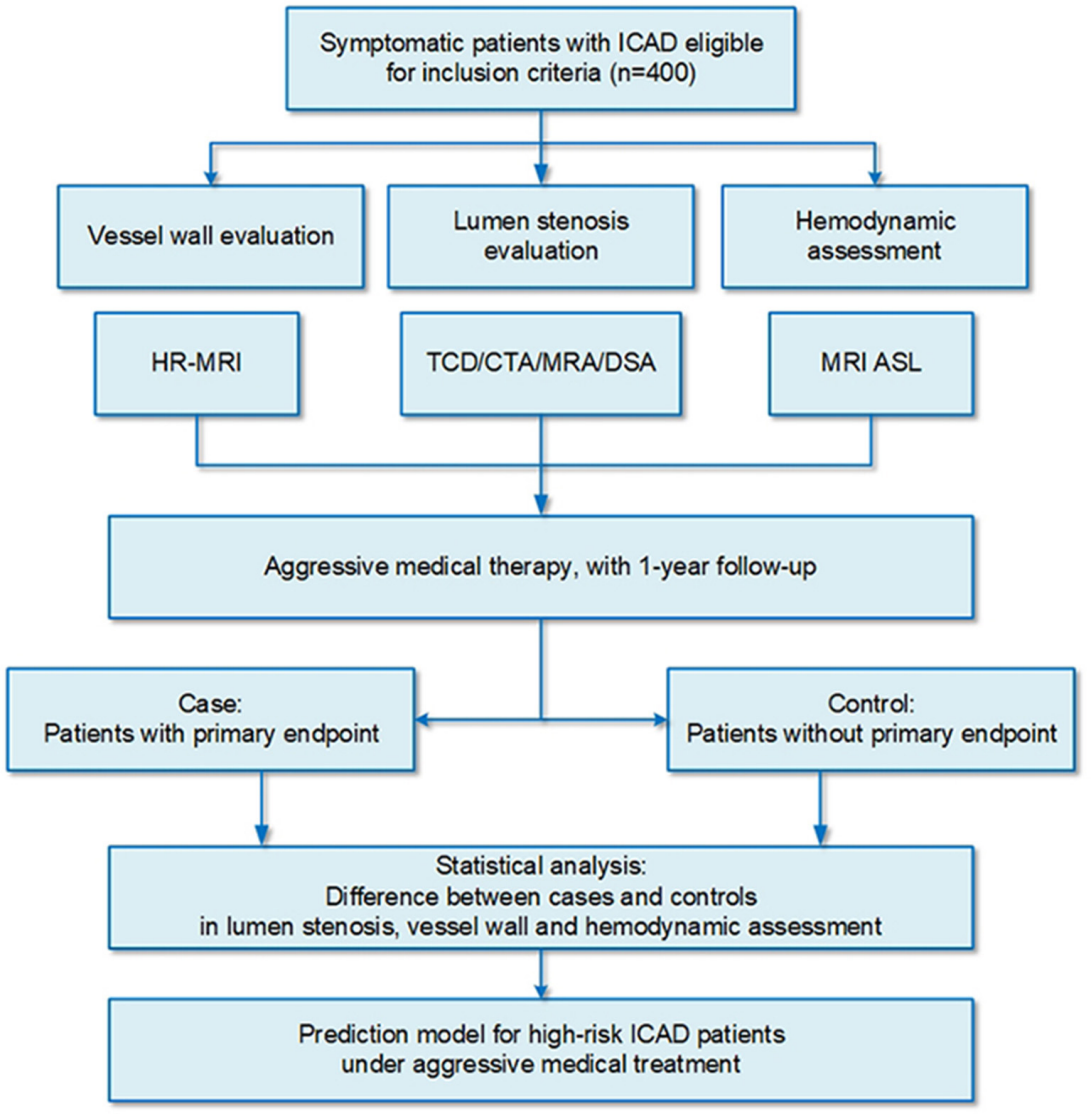
Schematic representation of the study.

This is a prospective, longitudinal, and nested case-control study of patients with ICAD who are treated with aggressive medical therapy in a tertiary hospital in northern China (Trial Registration No: ChiCTR2100048832) (Figure 1). This study aims to (i) identify multimodal imaging predictors of refractory to medical treatment of high-risk patients with ICAD and (ii) explore and validate a refined risk stratification model for patients with ICAD based on the above multimodal imaging predictors.

### Patient Selection

Patients diagnosed with symptomatic ICAD (≥ 50% stenosis) in the anterior circulation in our center will be enrolled in this study from December 2021 to November 2024. Participants will be required to sign informed consent prior to data collection and documentation. All patients will receive the cerebral artery imaging examination. Patients with more than 50% of stenosis in the extracranial carotid on the ipsilateral side will be excluded through transcranial Doppler ultrasound. Detailed inclusion and exclusion criteria are described in Table 1.

**Table 1:** Inclusion and exclusion criteria for patient selection.

**Inclusion criteria**	**Exclusion criteria**
1. Patients with symptomatic ICAS with culprit artery in the anterior circulation. 2. Patients aged from 18 to 80 years. 3. Stenosis of 50% to 99% (measured with WASID method) on TCD, CTA, or MRA initially, confirmed on DSA. 4. Informed consent is available from the patient or legal guardian.	1. Non-atherosclerotic lesion, for instance, moyamoya disease, vasculitis, vascular dissection, autoimmunity diseases, and developmental or genetic abnormalities. 2. More than 50% of stenosis in extracranial carotid on ipsilateral side. 3. Large cerebral infarction ( ≤ 1/2) identified by DWI and the baselin modified Rankin score of ≥3. 4. Antiplatelet drugs or statins are contraindicated. 5. MRI cannot be completed due to metal implants or claustrophobia.

### Study Procedures

#### Baseline Evaluations and Data Collection

All eligible patients are comprehensively evaluated from three aspects by non-invasive methods: lumen stenosis, plaque characteristics, and hemodynamics. Magnetic resonance angiography (MRA) is preferred to assess lumen stenosis at supplemented target lesion, while other examinations, such as transcranial Doppler ultrasound, CT angiography, or digital subtraction angiography, could be used as an alternative. The measurement of stenosis degree will be defined by the WASID criteria (15). The location and length of the lesion are measured. As for the assessment of plaque characteristics, HR-MRI (an imaging technique for wall visualization) is planned to perform with 3 Tesla and 3D sequences to assess the arterial remodeling index, plaque burden, and signal intensity. Hemodynamics is quantitatively evaluated by the MRI technology of artery spin labeling (ASL). Hypoperfusion will be defined as regional cerebral blood flow <90% of normal flow referring contralateral territory (31). Arterial transit artifact on ASL is defined as a focus or curvilinear hyperintensity located bordering the region of perfusion defects, which is a highly sensitive indicator of cerebral ischemia (32).

In addition, CFD simulation is applied to analyze the three-dimensional properties of the blood flow, including translesional pressure drop, wall shear stress, and filtration rate of low-density lipoprotein for a comprehensive evaluation of hemodynamic features. The above multimodal imaging examinations are necessary to carried out at the time of recruitment. All imaging parameters are described in Online Supplementary Appendix I.

#### Treatment Management

All participants are treated with optimal medical therapy according to the guidelines for ICAD from the American Heart Association/American Stroke Association, which indicates medical management, including antithrombotic therapy and vascular risk factor management, as the first-line therapy (8, 13). Antithrombotic therapies include anticoagulant and antiplatelet agents, which are recommended for all patients with ICAD without contraindications. Anticoagulant agents are recommended for cardiogenic embolism. Antiplatelet agents are more commonly used in patients with ICAD compared with anticoagulant agents. A 3-month dual antiplatelet therapy (e.g., aspirin 100 mg per day and clopidogrel 75 mg per day) is recommended for patients with early-arriving minor stroke and high-risk TIA, while it is not suitable for long-term treatment. Then, single antiplatelet therapy, including aspirin or clopidogrel, is applied throughout their lifetime for the prevention of secondary stroke in patients with ICAD. In addition, all patients with ICAD are required to follow the vascular risk factor management, which comprises the management of low-density lipoprotein (LDL-C), blood pressure, blood glucose level [fasting glucose level and hemoglobin A1c (HbA1c)], and lifestyle changes, such as Mediterranean diet and physical activity (see Table 2). Mediterranean diet is a low-fat diet emphasizing monounsaturated fat, plant-based food, and fish consumption with salt limitation of fewer than 6 grams per day (8). All medical management will be directed and monitored through the internet or telephone by investigators.

**Table 2:** The management of vascular risk factors and lifestyles.

**Risk factor and lifestyles**	**Target**
Blood pressure	<130/90 mmHg
LDL-c	<1.81 mmol/L (70 mg/dl)
Blood glucose	
Fasting	<5.9 mmol/L
HbA1c	<7%
Lifestyles	
Smoking cessation	Quitting smoking
Alcohol limitation	<2 times/week, <50 ml/time
Physical activity	Moderate-intensity aerobic activity more than 10 min 4 times a week or vigorous- intensity aerobic activity more than 20 min 2 times a week
Nutrition	Mediterranean-type diet with emphasis on a low-fat diet

#### Endpoint Evaluation

The primary endpoint of this study is be the ischemic stroke or death attributable to the lesion of the target vessel within 1 year after enrollment. The secondary endpoints are (1) any stroke or death; (2) all-cause mortality; (3) any stroke or death out of the territory of the responsible lesion, including hemorrhagic stroke, non-local ischemic stroke, and non-responsible vascular death; (4) functional outcome with the modified Rankin Scale (mRS).

Ischemic stroke is defined as the sudden onset of a new focal neurological deficit, such as hemiplegia, hemianopia, aphasia, dysphagia, sensory disorders, or confusion, lasting over 24 h, with new cerebral infarctions detected by MRI or CT. The classification of ischemic stroke for the responsible lesion is referred to as infarction location, as infarction in or out of the territory of the symptomatic intracranial artery. Hemorrhagic stroke is defined as the sudden onset of neurological deficits, with parenchymal, subarachnoid, or intraventricular hemorrhage detected by CT or MRI (33).

#### Follow-Up Assessment

All participants are followed up at 1 month, 6 months, and 1 year after enrollment on the internet or telephone (Table 3). We have established a follow-up system to direct and supervise the participants for better communication, feedback, and compliance. Unscheduled visits for any reason, as well as delayed visits within 7 days of the visit window, are recorded. If a participant reports ischemic symptoms, such as the sudden onset of disorder of motility or sensation, headache, dizziness, imbalance, epilepsy, and/or disorders of consciousness, we will arrange examinations of MRI or CT immediately for the patient and evaluate whether the therapy strategy should be altered. All cerebral arteries including extracranial and intracranial arteries are evaluated per patient at follow-up. During the follow-up period, the drug compliance evaluation scale are also be assessed.

**Table 3:** The planning of the follow-ups.

	**Screening period**	**Follow up period**			
**Visit time**	**Before intervention**	**1 m ±7 d**	**6 m ±7 d**	**12 m ±7 d**	**Unscheduled visit**
Informed consent	√				
Inclusion and exclusion criteria	√				
MRI DWI	√	√*	√*	√*	√
CTA/MRA/DSA	√				
MRI ASL	√				
mRS	√	√	√	√	√
Medical and medication history	√				
Medication	√	√	√	√	√
Intracranial ischemic event		√	√	√	√

√*The examination will be done when intracranial ischemic event occurs.

### Data Collection and Management

Data for patients enrolled in the study are collected through electronic case report forms. All de-identified data, including demographics, cerebrovascular events, lesion characteristics, and laboratory examination results, are collected by physicians at study entry and patient follow-up. All participants are expected to complete study visits over the planned 1-year follow-up period. In addition, this study is supervised by an independent Data Safety Monitoring Board (DSMB) that comprises independent experts from neurology, neuroradiology, and neurosurgery. The DSMB oversees the study by training and certificating the study staff, reviewing the general conduct of the trial, and checking the study data for quality and safety. Comprehensive training and regular assessment are carried out for all study staff to standardize data collection and assessment. The DSMB also gives suggestions for continuation, modification, or termination of the study. All adverse events that occurred during the study period are reported to the DSMB.

### Sample Size

By referring to the published study on medical management in patients with ICAD under 50 to 99% stenosis, the 1-year recurrence incidence of ischemic stroke is assumed to be 8.98% (5). The hazard ratio of hemodynamics (normal or compromised), which is a major risk factor for stroke, is assumed to be 2.4 (26). In addition, the loss rate of follow-up is assumed to be 10%. Thus, the study needs to recruit around 400 participants.

### Statistical Analysis

The results will be analyzed with the intention-to-treat method. All confounding factors that influenced outcomes will be analyzed, such as demographic data, cerebrovascular events, lesion characteristics, and laboratory examination results. Student *t*-test will be used for continuous variables, while the Mann-Whitney test will be used for ordinal variables. Count data of categorical variables will be analyzed by the Chi-square test or Fisher's exact probability method. *P* < 0.05 is considered statistically significant.

Subsequent multivariate analysis by logistic regression will be performed to detect independent relevant factors influencing primary endpoints. The regression coefficient of independent variables is defined as the weight of this variable for an evaluation system. If there are difficulties for building an evaluating system by the logistic regression model, we will attempt to establish the decision tree with high sensitivity for the evaluation system by Random Forest regression (34).

## Discussion

Intracranial atherosclerotic disease is the primary cause of stroke, especially in Asia, which caused high burdens of mortality and morbidity despite the latest treatment strategies, including medical management, endovascular treatment, and surgery (5). The effect of medical management seems ideal for a majority of patients with ICAD, while the current evaluation system cannot accurately predict those with a high risk of recurrent stroke under aggressive medical management (35). Although recurrent stroke is the multifocal cause in patients with symptomatic ICAD, studies have shown that the culprit lesion of ICAD was the major cause (36). Therefore, treatment for ICAD should be mainly focused on the culprit lesion. For the patients' refractory to medical management, other therapy strategies, such as endovascular treatment or surgery with direct intervention, has been adopted according to the current guideline recommendation (8, 13).

However, early diagnosis of high-risk patients' refractory to medical treatment and early triage to alternative therapy is limited by the current risk stratification system, which is merely based on the degree of luminal stenosis (37). The inclusion of the plaque characteristics and hemodynamics assessment with multimodal imaging method may provide a potential solution to make accurate risk stratification (22). To the best of our knowledge, the current study will be the first research to involve multimodal imaging evaluation from three aspects, including the degree of luminal stenosis, plaque characteristics, and hemodynamics, and plan to establish the prognosis prediction system for patients with ICAD who have medical treatment.

As an observational study, several potential limitations must be considered. The collected data may not be as robust as randomized control studies with blinding performance, while the randomized control studies may be needed to further confirm the results of the present study. Data collection and analysis will perform repeated verification and blinding assessment, respectively. Moreover, the study population will be focused on Chinese patients with ICAD; hence, caution must be applied when implementing the findings of this study in other ethnicities.

### Ethics and Dissemination

This study has been approved by the ethics committee of our center ([2021]083) and was prospectively registered (Registration No: ChiCTR2100048832). This study will be conducted according to the principles of the Declaration of Helsinki and subsequent amendments (29, 30). Study findings will be disseminated through peer-reviewed publications and presentations at scientific meetings.

## Ethics Statement

The studies involving human participants were reviewed and approved by the Ethics Committee of Xuanwu Hospital. The patients/participants provided their written informed consent to participate in this study.

## Author Contributions

YM and LJ: conceptualization. TW, JL, and CL: methodology and supervision. BY, RX, LL, KY, CZ, YW, YC, PG, and JC: data curation. TW and JL: project administration. CL: writing – original draft. All authors contributed to the article and approved the submitted version.

## Funding

This study was funded by the Beijing Science and Technologic Project (Z201100005520019). The funder was not involved in the study design, collection, analysis, interpretation of data, the writing of this article or the decision to submit it for publication.

## Conflict of Interest

CZ was employed by the company Shenzhen Escope Technology Co., Ltd., China. The remaining authors declare that the research was conducted in the absence of any commercial or financial relationships that could be construed as a potential conflict of interest.

## Publisher's Note

All claims expressed in this article are solely those of the authors and do not necessarily represent those of their affiliated organizations, or those of the publisher, the editors and the reviewers. Any product that may be evaluated in this article, or claim that may be made by its manufacturer, is not guaranteed or endorsed by the publisher.
