# Supplementary material for: High-Risk Intracranial Atherosclerotic Stenosis Despite Aggressive Medical Treatment: Protocol for a Prospective Nested Case-Control Study
Source: Front Neurol. 2022 Apr 13;13:803224. doi: 10.3389/fneur.2022.803224 (PMC9043759; doi:10.3389/fneur.2022.803224)
Supplement: Supplementary file 1 [file Data_Sheet_1.DOCX]

Appendix I. Parameterss of sequences on 3.0-T Siemens MR scanners

| Parameters | TOF | 3D-T1 | 3D-T2 | 3D-ASL |
| --- | --- | --- | --- | --- |
| TR(ms) | 22 | 900 | 1200 | 4000 |
| TE(ms) | 3.74 | 20 | 110 | 19.82 |
| FOV(mm^2^) | 200×175 | 200×158 | 180×180 | 192×192 |
| Matrix | 240×320 | 320×252 | 272×320 | 126×128 |
| Slice thickness(mm) | 0.6 | 0.7 | 0.6 | 3 |
| Flip angle (°) | 18 | - | - | 180 |
| Echo train(ms) | - | 356 | 246 | - |
| Bandwidth(Hz/pixel) | 186 | 326 | 401 | 2112 |
| Scan time | 4 min 55s | 9 min 53s | 7 min 54s | 4 min 52s |

TOF, time of flight; T1, T1-weighted imaging; T2, T2-weighted imaging; ASL, arterial spin labeling; TR, repetition time; TE, echo time; FOV, field of view
